# The yeast enzyme Eht1 is an octanoyl-CoA:ethanol acyltransferase that also functions as a thioesterase

**DOI:** 10.1002/yea.3046

**Published:** 2014-11-04

**Authors:** Michael J Knight, Ian D Bull, Paul Curnow

**Affiliations:** 1 School of Biochemistry, University of BristolUK; 2 School of Chemistry, University of BristolUK

**Keywords:** enzyme kinetics, protein purification, protein expression, medium chain fatty acid ethyl ester, coenzyme A

## Abstract

Fatty acid ethyl esters are secondary metabolites that are produced during microbial fermentation, in fruiting plants and in higher organisms during ethanol stress. In particular, volatile medium-chain fatty acid ethyl esters are important flavour compounds that impart desirable fruit aromas to fermented beverages, including beer and wine. The biochemical synthesis of medium-chain fatty acid ethyl esters is poorly understood but likely involves acyl-CoA:ethanol *O*-acyltransferases. Here, we characterize the enzyme ethanol hexanoyl transferase 1 (Eht1) from the brewer's yeast *Saccharomyces cerevisiae*. Full-length Eht1 was successfully overexpressed from a recombinant yeast plasmid and purified at the milligram scale after detergent solubilization of sedimenting membranes. Recombinant Eht1 was functional as an acyltransferase and, unexpectedly, was optimally active toward octanoyl-CoA, with *k_cat_* = 0.28 ± 0.02/s and *K_M_* = 1.9 ± 0.6 μm. Eht1 was also revealed to be active as a thioesterase but was not able to hydrolyse *p*-nitrophenyl acyl esters, in contrast to the findings of a previous study. Low-resolution structural data and site-directed mutagenesis provide experimental support for a predicted *α*/*β*-hydrolase domain featuring a Ser–Asp–His catalytic triad. The *S. cerevisiae* gene YBR177C/*EHT1* should thus be reannotated as coding for an octanoyl-CoA:ethanol acyltransferase that can also function as a thioesterase. © 2014 The Authors. Yeast published by John Wiley & Sons, Ltd.

## Introduction

The ubiquitous industrial yeast *Saccharomyces cerevisiae* produces fatty acid ethyl esters (FAEEs) as secondary metabolites during fermentation. The volatile medium chain-length FAEEs (C4–C12) are of interest because they make an important contribution to the flavour profile of fermented beverages such as beer and wine (Saerens *et al.*, 2010; Robinson *et al.*, 2014). Understanding the biochemistry of medium-chain FAEEs may thus offer a route to understanding and manipulating the flavour of yeast-derived beverages, which have substantial economic and cultural value. For example, the EU alone produces nearly 40 billion litres of beer annually, with a sales value of over €100 billion (Berkhout *et al.*, 2013).

Yeast FAEE biosynthesis likely proceeds enzymatically via a family of acyl-CoA:ethanol *O*-acyltransferases (AEATases) that catalyse the transfer of fatty acyl groups from acyl-CoA to ethanol (Malcorps and Dufour, 1992; Mason and Dufour, 2000; Saerens *et al.*, 2006). This generates medium-chain FAEEs including ethyl hexanoate, ethyl octanoate and ethyl decanoate, which have pleasant apple-like, aniseed and floral aromas, respectively. Four lipoproteins from *S. cerevisiae* have been identified as being responsible for the synthesis of short- and medium-chain aliphatic esters. These could be partially purified and an *n*-hexanoyl-CoA:ethanol-*O*-acyltransferase, subsequently named Eht1, was identified with *K_M_* toward hexanoyl-CoA of approximately 18 μm (Mason and Dufour, 2000). *Eht1* knock-out strains are viable but temperature-sensitive (Athenstaedt *et al.*, 1999), with an extended lag phase in culture growth (Saerens *et al.*, 2006).

A putative family of medium-chain AEATases in *S. cerevisiae* was subsequently described based upon amino acid sequence homology with Eht1 (Saerens *et al.*, 2006). This group comprises open reading frames YBR177C (common gene name *EHT1*; gene product Eht1), YPL095C (*EEB1*; Eeb1) and YMR210W (no common name for gene or gene product). Pairwise alignment suggests that Eht1 and Eeb1 are closely related, with 58% sequence identity and 73% sequence similarity at the amino acid level. This is consistent with these two proteins being paralogues that arose from gene duplication during the *S. cerevisiae* genome duplication (Byrne and Wolfe, 2005). YMR210w is more divergent, being 29% identical and 43% similar to Eht1 and 28% identical and 44% similar to Eeb1. A BLAST search reveals that putative AEATases with related sequences occur relatively widely among other fungi.

There are conflicting reports on whether the knock-out or overexpression of AEATases in general, and Eht1 in particular, can influence FAEE production in *S. cerevisiae* during fermentation (Lilly *et al.*, 2006; Saerens *et al.*, 2006, 2008; Rossouw *et al.*, 2008; Chen *et al.*, 2014) and such studies are likely compromised by metabolic redundancy (Saerens *et al.*, 2010). In particular, Saerens and colleagues studied the role of Eht1, Eeb1 and Ymr210w in FAEE synthesis by the complementary methods of combinatorial gene deletion and overexpression after genomic integration (Saerens *et al.*, 2006). Deletion strain *eht1Δ* produced 36% less ethyl hexanoate (C6) and 26% less ethyl octanoate (C8) relative to wild-type, while the synthesis of ethyl butanoate (C4) was unaffected and the synthesis of ethyl decanoate (C10) slightly increased. The mutation *eeb1Δ* had a greater impact on FAEE production, reducing the synthesis of each of ethyl butanoate, ethyl hexanoate, ethyl octanoate and ethyl decanoate by 36%, 88%, 45% and 40% relative to wild-type. Deletion strain *ymr210wΔ* showed no changes in FAEE synthesis. A double mutant, *eht1Δ*/*eeb1Δ*, gave results similar to *eeb1Δ* alone. The authors concluded that Eeb1 was the major protein governing FAEE synthesis in *S. cerevisiae*, with Eht1 playing a minor role and YMR210W being irrelevant. Surprisingly, the overexpression of Eht1 and Eeb1 did not result in increased ethyl ester synthesis, although an increase in ethyl hexanoate was reported when yeasts overexpressing Eht1 were engineered to provide increased levels of the precursor hexanoyl-CoA. Cellular availability of the relevant precursors is thus thought to be a critical limiting factor in ethyl ester production by the AEATases (Saerens *et al.*, 2008). It has also been proposed that the AEATases may have homeostatic esterase activity that prevents the accumulation of high concentrations of FAEEs (Saerens *et al.*, 2006).

Recombinant Eeb1 and Eht1 were also previously purified from *Escherichia coli* as glutathione-*S*-transferase fusion proteins (Saerens *et al.*, 2006). The purified recombinant enzymes appeared to function as AEATases, as monitored by GC–MS. However, both Eeb1 and Eht1 had surprisingly low acyltransferase activities of 1–3 nm/min/mg and the apparent chain length preference of the recombinant proteins did not correlate with the results from the deletion strains in the same paper. This report also measured the esterase activity of these purified enzymes against *p*-nitrophenyl esters. Interestingly, the chain length preferences for esterase activity were only partly consistent with the transferase activity and gene knock-outs. For example, strain *eeb1Δ* showed a reduction in FAEEs with chain-length preference C6 > > C8 > C10 > C4, but recombinant Eeb1 had a preference of C8 > > C6 > C4 for ester synthesis and C2 > C4 > C6 > C8 > C10 for esterase activity.

It is not clear that *E. coli* provides an ideal expression host for Eht1, which is a lipoprotein localized to the yeast lipid particle and mitochondria (Malcorps and Dufour, 1992; Athenstaedt *et al.*, 1999; Zahedi *et al.*, 2006). To overcome this issue and to further study the activity of the AEATases *in vitro*, we here develop methods to isolate and characterize recombinant AEATases after plasmid-based expression in a yeast host. This includes novel detergent-based methods for protein purification and the introduction of a coupled enzyme assay to study protein function.

## Materials and methods

### Materials

Enzymes for molecular biology were from New England Biolabs. TOP10 ultracompetent cells, the pYES2CT vector, anti-V5-HRP, molecular weight markers and native PAGE gels and reagents were from Life Technologies. Glass econocolumns and the DC protein assay kit were from BioRad. Denatured sheared salmon sperm DNA and reagents for assays of esterase and acyltransferase activity were from Sigma, except *p*-nitrophenyl hexanoate from Ark chemicals. HisTrap columns, PD-25 gel filtration columns, size-exclusion columns, protein chromatography standards and nitrocellulose membrane were from GE Healthcare. Precast acrylamide gels were from NuSep. LumiGLO chemiluminescence reagents were from Cell Signaling Technology.

### Gene cloning

Genomic DNA from *Saccharomyces cerevisiae* FGY217 (*ura3-52*, *pep4Δ*) (Kota *et al.*, 2007) was isolated from cell lysate by ethanol precipitation. DNA was judged to be of acceptable purity, with *A*_260_/*A*_280_ = 2.3. The open reading frames YBR177C (gene name *EHT1*), YPL095C (*EEB1*) and YMR210W (no common name) were amplified without stop codons from the gDNA template by PCR. Non-complementary sequences corresponding to unique restriction sites were included at the 5′-ends of both the forward and reverse primers to facilitate cloning. The forward and reverse primers for *EHT1* were 5′-ATGCGGATCCAATGTCAGAAGTTTCCAAATGG-3′ and 5′-ATGCTCTAGATACGACTAATTCATCAAAC-3′, respectively, flanking the gene with sites for restriction enzymes *Bam*HI and *Xba*I; for *EEB1*, 5′-CATCAAAGCTTATGTTTCGCTCGGGTTAC-3′ and 5′-ATCAGGATCCCCTAAAACTAACTCATCAAAGC-3′ (*Hin*dIII/*Bam*HI); and for YMR210W, 5′-CATCAAAGCTTATGCGTCTAAAAGAATTGTTACC-3′ and 5′-CATCAGAGCTCCCATTCGCGCGAAAGGTTGTGG-3′ (*Hin*dIII/*Sac*I). Full-length PCR products were separated from primers and partial products on a 1% agarose gel in TBE buffer and recovered.

PCR products were digested with the relevant enzymes to generate cohesive ends and ligated into the similarly digested yeast shuttle vector pYES2CT. For *EHT1*, pYES2CT was *Bam*HI/*Xba*I-digested and the small fragment excised from the multiple cloning site was removed by gel electrophoresis as above. For *EEB1* and YMR210W, the *Bam*HI/*Xba*I-digested vector was blunted and the resulting blunt ends ligated. This procedure destroys the *Xba*I site and places the *Hin*dIII, *Sac*I and *Bam*HI sites at the furthest downstream end of the multiple cloning region. This modified vector was then used for cohesive end ligation after restriction digest. Genes were cloned in-frame with downstream vector sequences for a V5 epitope (amino acid sequence GKPIPNPLLGLDST), a His_10_ purification tag and a stop codon. Site-directed mutagenesis of Eht1 to generate S247A and D395N was carried out via the commercial ‘Quikchange’ procedure (Agilent). All constructs were verified by sequencing.

### Protein expression and purification

Transformed yeast cell lines were grown in selective broth (– uracil) with 2% glucose. For protein expression cells were harvested, resuspended in a small volume and used to inoculate 1 l cultures of selective broth supplemented with 2% galactose and 0.1% glucose to a final *A*_600_ of 0.4. Induction cultures were grown for 24 h at 30 °C with constant agitation at 230 rpm.

Expression cultures were harvested at 3300 × *g* and resuspended in 50 ml phosphate-buffered saline (PBS). The cells were lysed at 35 KPSI in a cell disrupter (Constant Systems) and unbroken cells were pelleted at 5000 × *g*. The lysate was clarified by pelleting the cell membranes at 150 000 × *g* for 1 h. The membrane pellet was resuspended in 50 mm sodium phosphate, pH 7.4, 150 mm NaCl, 5% glycerol, 100 mm sucrose at a total protein concentration of 4 mg/ml and subject to at least 30 passes in a homogenizer. The membranes were solubilized by the addition of 1–2% fos-choline-12 (FC12; also known as *n*-dodecylphosphocholine) for 60 min at 4 °C.

Proteins were purified on a Ni-NTA affinity resin under stringent conditions, with imidazole maintained at 20–50 mm in all buffers to prevent background binding. A slurry of Ni-NTA resin of 2 ml column volume was equilibrated in Column Buffer (50 mm sodium phosphate, pH 7.4, 150 mm NaCl, 5% glycerol, 0.1% FC-12) plus 20 mm imidazole. The equilibrated slurry was incubated with solubilized membranes plus 20 mm imidazole for 90 min at 4 °C. The resin was either allowed to settle under gravity or pelleted at 800 × *g* for 3 min and the supernatant, containing unbound protein, was removed. The resin was washed five times with 20 ml column buffer plus 50 mm imidazole. After the final wash, the bead slurry was resuspended in a small volume of column buffer plus 50 mm imidazole and poured into a glass column. The buffer was removed at a flow rate of 1 ml/min and protein was eluted from the column in 10 ml column buffer with 0.5 m imidazole at a flow rate of 0.2 ml/min. The eluent was immediately concentrated and passed through a PD-25 desalting column to remove imidazole. The IMAC-purified proteins were either used directly or subjected to further purification by size-exclusion chromatography.

### Protein analysis

Size exclusion chromatography (SEC) was performed with a Superdex 200 10/300 GL column. The column was equilibrated in Column Buffer before 1 ml samples were loaded and run at 0.5 ml/min. Molecular weight standards were run in the same buffer for calibration.

SDS–PAGE was performed with precast 12% Tris–glycine gels. For western blotting, transfer to nitrocellulose membranes was performed in 12 mm Tris, 96 mm glycine, 20% methanol and 0.01% SDS, pH 8.3. After blocking with PBS containing 0.1% Tween and 5% low-fat dried milk powder, the recombinant V5 epitope was probed with mouse monoclonal anti-V5 conjugated with horseradish peroxidase. Chemiluminescence was detected on photographic film.

Non-denaturing (native) PAGE was performed on 4–16% acrylamide Bis–Tris gels with commercial reagents, according to the manufacturers' instructions. Coomassie G-250 was added to samples at 0.005% before loading.

Total protein concentration in cell fractions was determined using a detergent-compatible Lowry assay or by absorbance at 280 nm using a calculated extinction coefficient (www.expasy.org/protparam) of 75 080 /m/cm for Eht1.

Circular dichroism was carried out on an Aviv instrument in a 0.5 mm path-length cell at a protein concentration of 0.65–0.9 mg/ml. The approximate *α*-helical content was calculated using equation (1) (Morrow *et al.*, 2000; MacRaild *et al.*, 2001): (1)
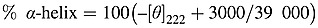


### Esterase activity

Purified Eht1 was incubated at 25 °C with 50 μm *p*-nitrophenylbutyrate, *p*-nitrophenylhexanoate, *p*-nitrophenyloctanoate, *p*-nitrophenyldecanoate or *p*-nitrophenyldodecanoate in 50 mm sodium phosphate buffer, pH 7.4, 150 mm NaCl. Esterase activity leading to the liberation of *p*-nitrophenol was monitored by absorbance of the sample at 400 nm relative to a standard curve.

### Acyltransferase activity

A coupled assay (Dunn *et al.*, 2013) was used to monitor acyltransferase activity at 25 °C. Typically, 1–4 µl purified enzyme at 1.5 mg/ml was incubated with 400 µl assay buffer, comprising 50 mm sodium phosphate, pH 7.4, 0.4 mm nicotinamide adenine dinucleotide (NAD^+^), 0.4 mm thiamine pyrophosphate (TPP), 2 mm *α*-ketoglutarate, 1 mm EDTA, 0.4 U *α*-ketoglutarate dehydrogenase, 0.125% ethanol, and acyl-CoA at various concentrations as required. Liberation of CoA by AEATase activity was thus coupled to the generation of NADH, and could be monitored via absorbance at 340 nm or by fluorescence with excitation at 340 nm and emission at 460 nm, with identical results. Data were fit to a hyperbole through non-linear regression with GraphPad Prism, assuming a monomer active unit in all calculations.

### Gas chromatography–mass spectrometry (GC–MS)

A modified solid phase microextraction (SPME) procedure was used to enable analysis by GC–MS (Plutowska and Wardencki, 2008). The reaction mixture was 125 μm acyl-CoA substrate, 5% ethanol and 560 nm Eht1 in 50 mm sodium phosphate buffer, pH 7.4. After 30 min of incubation at room temperature, 200 µl of the reaction mixture was transferred into a 10 ml glass vial and a conditioned polydimethylsiloxane (PDMS) fibre (Supelco; 100 µm) was introduced into the vial headspace. After 30 min at 30 °C, the fibre was removed from the vial and immediately inserted into the injection port of a ThermoQuest TraceMS instrument fitted with a 50 m × 0.32 mm × 0.17 mm HP1 column (Agilent Technologies). The injector was maintained at a constant 240 °C. The oven temperature was initially held at 40 °C for 1 min before being increased to 180 °C at a rate of 5 °C/min, then to 240 °C at a rate of 10 °C/min with a final hold time of 13 min. Samples were analysed under electron ionization conditions, with the MS scanning between *m/z* 50–650. Ethyl esters were identified based upon their characteristic mass spectra and related to a library of known alkyl esters (Christie, 2014).

## Results

### Recombinant expression of Eht1, Eeb1 and YMR210w

**Figure 1:**
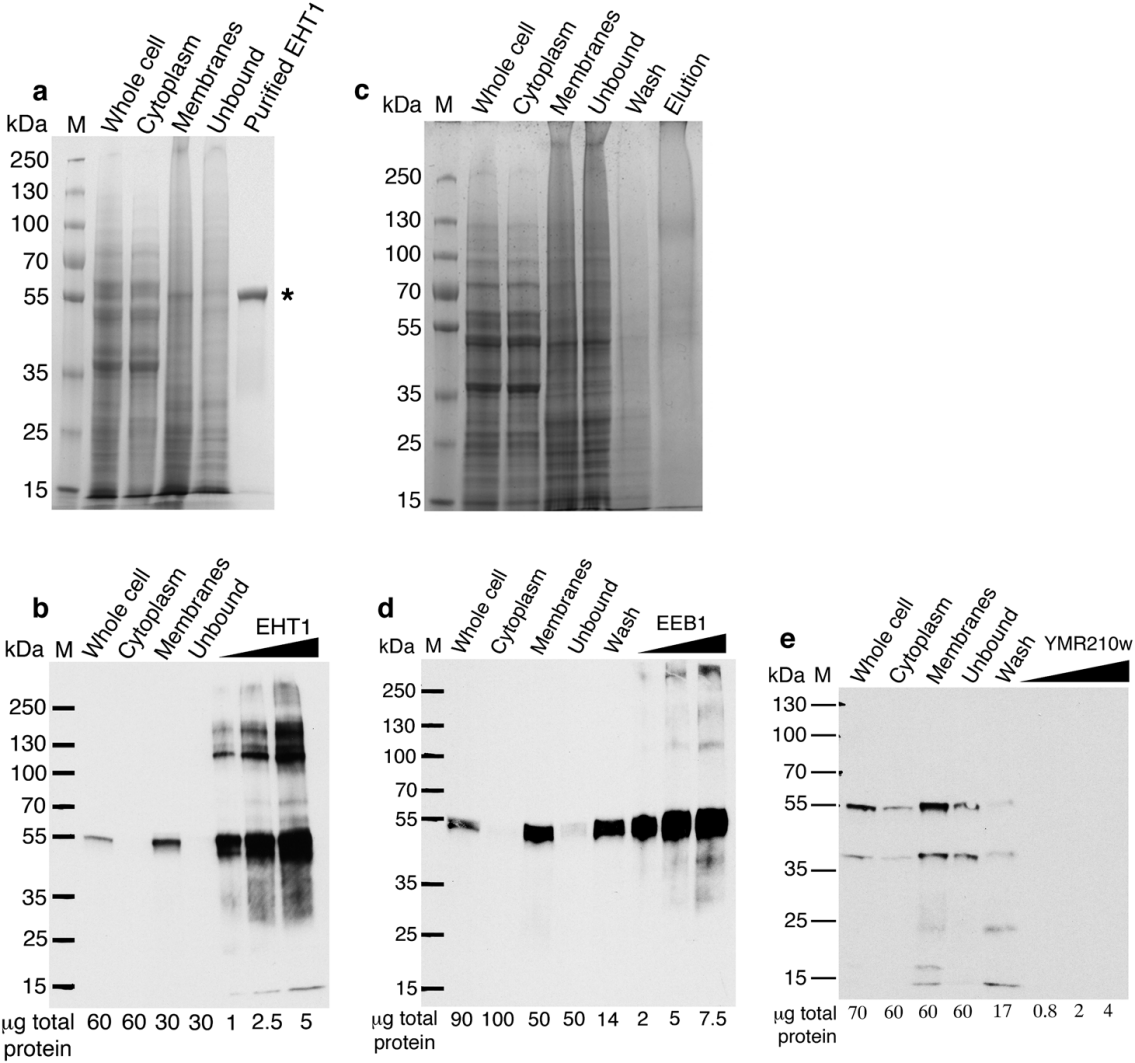
Expression and purification of yeast AEATases. (a) Recombinant Eht1 was purified from the sedimenting membrane fraction of induced yeast cells. Purified Eht1 ran at the expected molecular weight of 55 kDa on a Coomassie-stained SDS–PAGE gel (*). (b) Western blotting of Eht1 cell fractions under rapid exposure confirmed that Eht1 was expressed into sedimenting membranes. (c, d) Eeb1 was expressed into sedimenting membranes at low levels only and could not be visualized on a Coomassie-stained gel. (e) Western blotting of YMR210w purification fractions indicated membrane localization similar to Eht1 and Eeb1 but showed multiple bands suggesting proteolysis. M, molecular weight markers in kDa as shown; lane headings are relevant to cell fractionation (whole cell, cytoplasm, membrane) or affinity purification (unbound, wash, elution or protein name)

We attempted to express each of Eht1, Eeb1 and Ymr201w with a polyhistidine tag to allow protein purification by immobilized metal affinity chromatography (IMAC). Figure1 compares the expression and purification of Eht1 and Eeb1 followed by Coomassie-stained SDS–PAGE (Figure1a, c) and western blotting (Figure1b, d, e). Western blotting of cell fractions determined that Eht1 was localized to sedimenting membranes (Figure1b), consistent with cellular localization into the yeast lipid particle and mitochondria (Malcorps and Dufour, 1992; Athenstaedt *et al.*, 1999; Zahedi *et al.*, 2006). These membranes were solubilized with the detergent FC12 and successfully purified on an immobilized nickel column. Purified Eht1 was visualized by Coomassie staining as a single band close to the theoretical molecular weight of 55 kDa (Figure1a). Eht1 purification yields were typically 0.7 mg purified protein/l yeast culture. In contrast, purified Eeb1 could not be visualized by Coomassie (Figure1c), although western blotting confirms that the protein is overexpressed at low levels, localizes to sedimenting membranes, binds to the IMAC column and is enriched in the column eluate (Figure1d). Figure1d was subject to extensive exposure in order to achieve an image contrast similar to Figure1b, and the band intensities are not directly comparable. Recombinant Eeb1 is thus expressed only at low levels that are unsuitable for biochemical analysis. Ymr210w was expressed into sedimenting membranes at low levels and apparently proteolysed, appearing as multiple bands on a long-exposure western blot (Figure1e), and was not pursued further. For unknown reasons, it thus appears that recombinant Eeb1 and YMR210W are subject to tight control over their expression within the cell, but that Eht1 can be overexpressed within the yeast host. This is in agreement with proteomics data (Table1) suggesting that Eht1 is substantially the most abundant of the AEATases within the yeast proteome.

**Table 1:** Aggregate proteomics data for yeast AEATases: cellular abundance in ppm from different proteomics studies after searching the PaxDb *S. cerevisiae* dataset (Wang *et al.*, 2012) with AEATase gene names

	*S. cerevisiae* cellular abundance (ppm)		
Source	Eht1	Eeb1	Ymr210w
www.peptideatlas.org	172	–	0
Newman *et al.*, 2006	81/186	–	–
www.thegpm.org	310	3	79
de Godoy *et al.*, 2008	209	0	3
Ghaemmaghami *et al.*, 2003	57	13	–
Lu *et al.*, 2007	86	–	–
PaxDb integrated dataset (Wang *et al.*, 2012)	113	0	2

–, protein absent from dataset.

### Oligomeric state of recombinant Eht1

**Figure 2:**
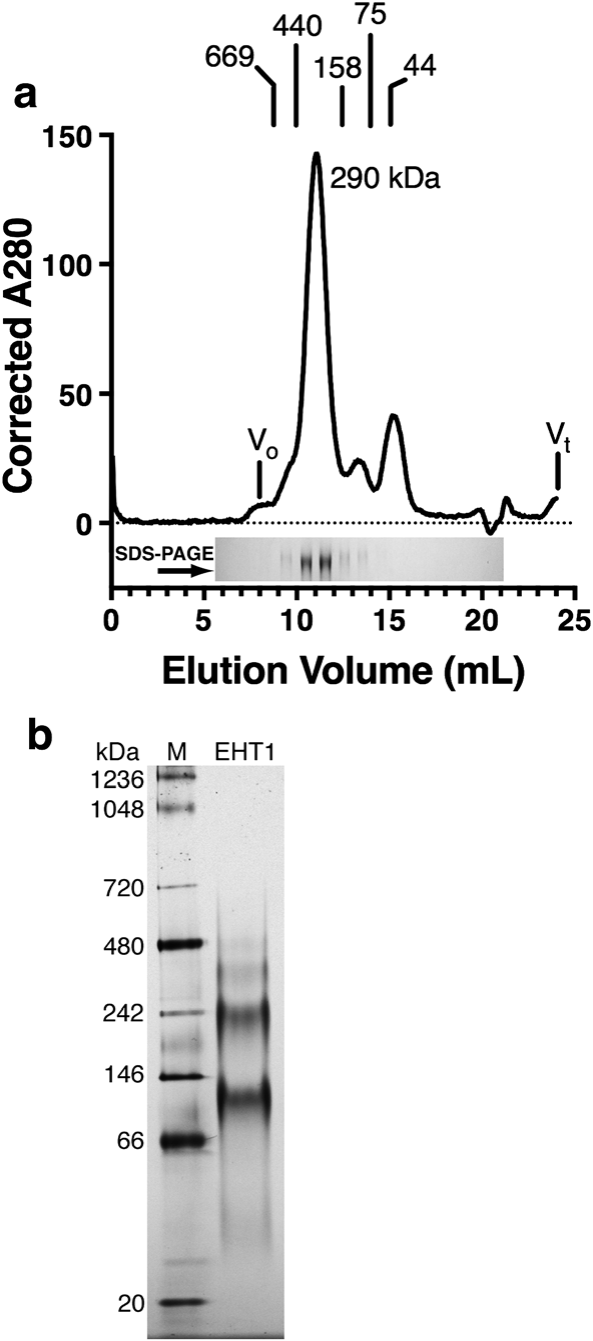
Characterization of purified Eht1. (a) Size-exclusion chromatogram of detergent-solubilized Eht1 consistent with an apparent homopentamer, relative to protein standards as shown. Fractions were collected and applied to an SDS–PAGE gel to confirm the peak identity as Eht1 and to verify that that no other proteins were co-purified. (b) Eht1 exhibits multiple bands on blue native PAGE

Size-exclusion chromatography of purified recombinant Eht1 resolved a single, monodisperse peak at an apparent molecular weight of 290 kDa relative to molecular mass standards (Figure2a). The identity of the peak was confirmed to be Eht1 by analysing the peak fractions with SDS–PAGE, and no bands other than that corresponding to the Eht1 monomer were observed. Given that the theoretical mass of recombinant Eht1 is 55 kDa, Eht1 in FC12 has a size exclusion profile consistent with being a homopentamer. Figure2b shows that Eht1 exhibited complex behaviour on blue native PAGE gels, running as two major bands at 120 and 225 kDa; these bands approximate to the theoretical sizes of dimer and tetramer, respectively. Purified Eht1 thus exhibits reversible oligomerization in FC12. This phenomenon presumably arises from non-specific hydrophobic interactions, perhaps mediated by hydrophobic protein surfaces that are normally accommodated by the lipid particle. The oligomerization is apparently reversible and can be disrupted by the binding of, for example, Coomassie blue to protein hydrophobic patches on blue native PAGE (Figure2b). It is not clear whether such oligomerization is physiologically relevant.

### Secondary structure of Eht1

**Figure 3:**
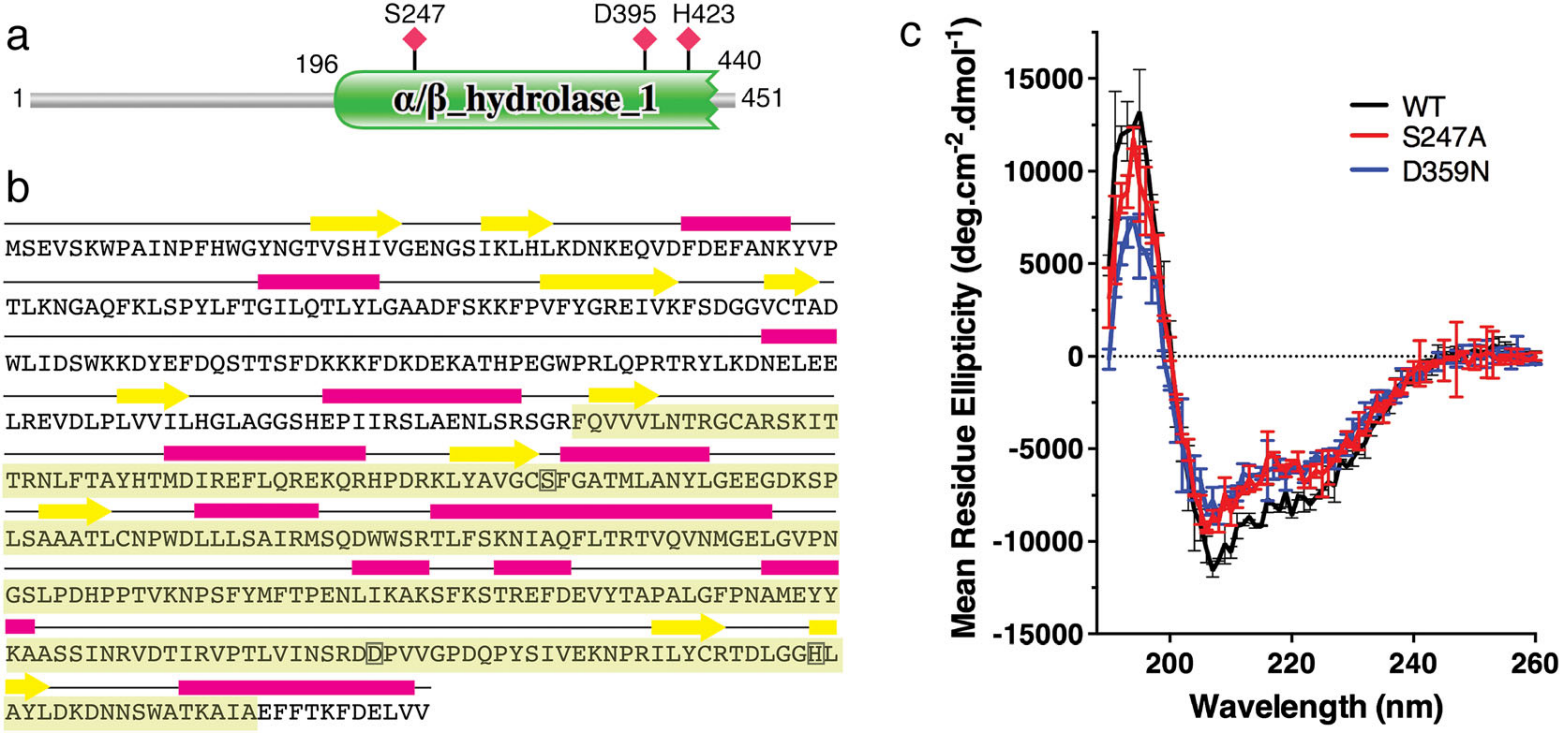
Secondary structure of Eht1. (a) Multiple sequence alignment predicts an *α*/*β*-hydrolase domain at the Eht1 C-terminus that contains the core catalytic residues S247, D395 and H423. (b) The predicted mixed *α*/*β* secondary structure of Eht1. The hydrolase domain is shaded green and active site residues are boxed. (c) The predicted secondary structure composition was confirmed by circular dichroism measurements. Data are mean ± SD from two replicates

Multiple sequence alignment predicts an *α*/*β*-hydrolase domain at the Eht1 C-terminus that contains the core catalytic residues S247, D395 and H423 (http://pfam.xfam.org; and Saerens *et al.*, 2006). This is shown schematically in Figure3a. The N-terminal region 1–196 does not have significant homology to any known protein fold. However, Figure3b shows the results of sequence analysis by the PSIPRED server (http://bioinf.cs.ucl.ac.uk/psipred/), which predicts substantial secondary structure in the N-terminal region of the protein. This was confirmed by circular dichroism measurements (Figure3c). The circular dichroism spectra were characteristic of mixed *α*/*β* structure with approximately 25% overall *α*-helical content. Figure3c also confirms that site-directed mutagenesis at the active site residues S247A and D395N had no effect on the overall protein structure.

### Eht1 assay by GC–MS

**Figure 4:**
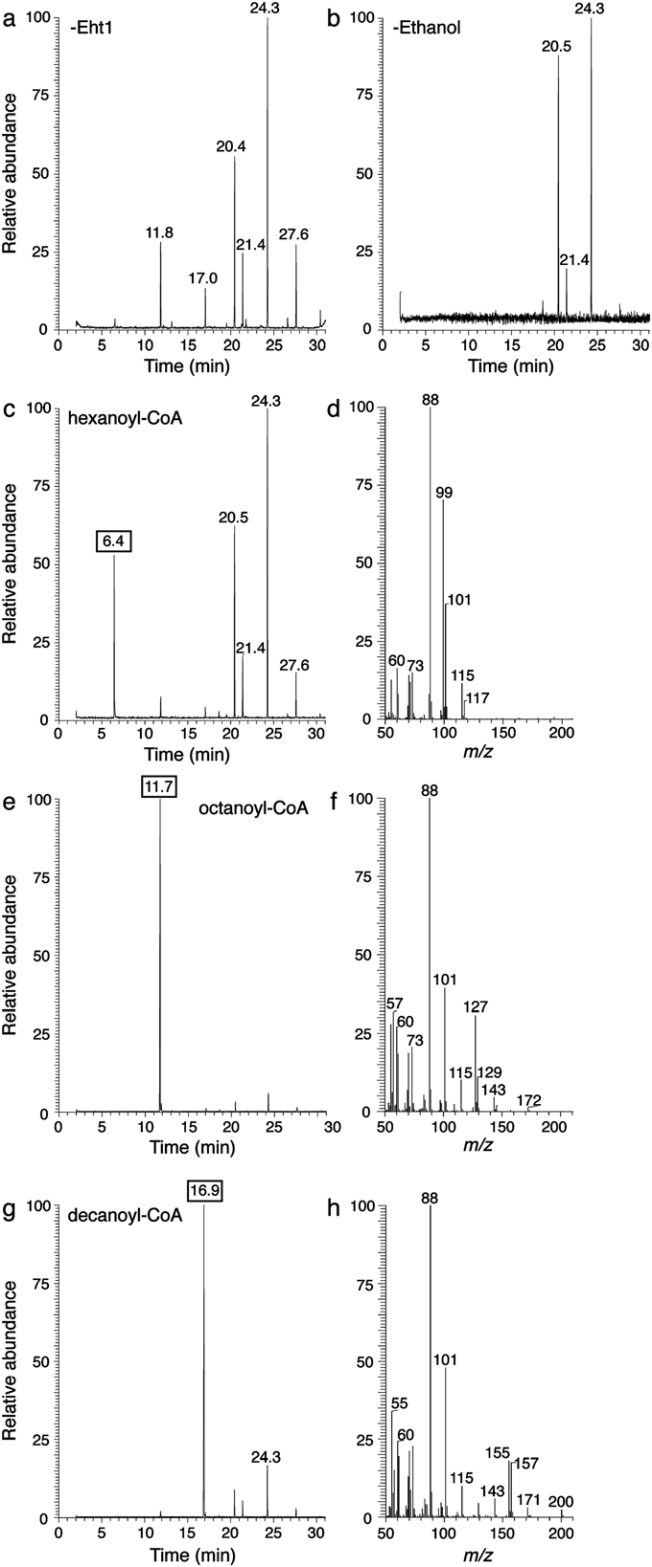
GC–MS of medium-chain FAEEs synthesized by Eht1 from acyl-CoA and ethanol. (a) Controls without Eht1 and (b) without ethanol show buffer background peaks. (c) In the full-treatment experiment combining Eht1, ethanol and hexanoyl-CoA, a modest GC peak was observed at 6.4 min. (d) MS of the 6.4 min peak gave the expected fragments for ethyl hexanoate (M^+•^, expected *m/z* = 144, weak signal; McLafferty rearrangement, *m/z* = 88; loss of ethoxide ion, *m/z* = 99; loss of ethyl group, *m/z* = 115; C_3_H_5_O_2_, *m/z* = 73; C_5_H_9_O_2_, *m/z* = 101). (e) GC and (f) MS of ethyl octanoate synthesized enzymatically from octanoyl-CoA and ethanol. The intense 11.7 min peak gave the expected fragments for ethyl octanoate (M^+•^, *m/z* = 172; McLafferty rearrangement, *m/z* = 88; loss of ethoxide ion, *m/z* = 127; loss of ethyl group, *m/z* = 143; C_3_H_5_O_2_, *m/z* = 73; C_5_H_9_O_2_, *m/z* = 101; C_6_H_11_O_2_, *m/z* = 115; C_7_H_13_O_2_, *m/z* = 129). (g) GC and (h) MS of ethyl decanoate synthesized enzymatically from decanoyl-CoA and ethanol. MS of the 16.9 min peak is characteristic of ethyl decanoate (M^+•^, *m/z* = 200; McLafferty rearrangement, *m/z* = 88; loss of ethoxide ion, *m/z* = 155; loss of ethyl group, *m/z* = 171; C_5_H_9_O_2_, *m/z* = 101; C_6_H_11_O_2_, *m/z* = 115)

We next assessed the function of purified Eht1 through qualitative gas chromatography–mass spectrometry (GC–MS). Eht1 was indeed active in synthesizing FAEEs that could be clearly resolved and unambiguously assigned by GC–MS. Figure4 shows the synthesis of FAEEs by Eht1 from acyl-CoA and ethanol. Negative controls were carried out in the presence of octanoyl-CoA but without Eht1 (Figure4a) or without ethanol (Figure4b). Major buffer background peaks were observed in these controls at retention times of 11.8, 20.5, 21.4, 24.3 and 27.6 min. In the presence of Eht1 (Figure4e), a single additional peak was seen with a retention time of 11.7 min. This peak was identified by MS as ethyl octanoate (Figure4f). The molecular ion (M^+•^) was observed at *m/z* = 172 and the diagnostic ion from the McLafferty rearrangement of an ethyl ester is observed at *m/z* = 88. Peaks corresponding to the loss of ethoxide ion at [M-45]^+^ and the loss of the ethyl group at [M-29]^+^ were present, as well as the expected aliphatic chain fragments (C*_n_*H_2*n*-1_O_2_). Eht1 was able to synthesize ethyl hexanoate from hexanoyl-CoA and ethanol (Figure4c, d) but with a qualitatively lower yield than either ethyl octanoate (Figure4e) or ethyl decanoate (Figure4g, h). This is in agreement with enzymatic assays below (Figure6) suggesting that octanoyl-CoA is the preferred substrate for Eht1.

### Biochemical assay of Eht1 activity

**Figure 5:**
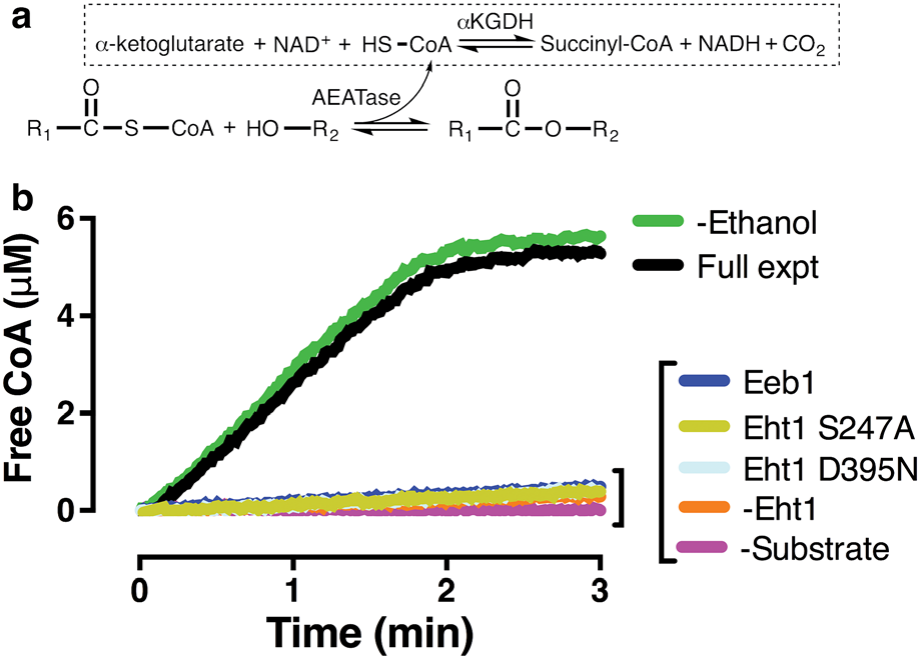
Coupled enzymatic assay for Eht1 activity. (a) General scheme of the AEATase reaction coupled to the activity of *α*-ketoglutarate dehydrogenase (*α*KGDH). (b) Turnover of octanoyl-CoA by Eht1 monitored by the coupled assay. All experiments are at 0.14 μm protein, 0.125% ethanol, 10 μm octanoyl-CoA (except for Eeb1; see below). The assay is sensitive to the presence of the enzyme and the acyl-CoA substrate but, unexpectedly, is insensitive to ethanol (see main text for discussion). The Eht1 active site mutants *S247A* and *D395N* were inactive, similar to experiments conducted in the absence of protein (–*Eht1*) and the absence of substrate (–*Substrate*). Semi-purified Eeb1 was inactive, as shown against 62.5 μm octanoyl-CoA and against the other acyl-CoA substrates tested at all concentrations

Dunn *et al.* (2013) described a continuous coupled assay that was used to study the acyltransferase domain of a polyketide synthase. The principle of this assay is that the transfer of acyl chains from acyl-CoA to ethanol liberates CoA. This free CoA is used as a co-substrate for *α*-ketoglutarate dehydrogenase, causing the reduction of NAD^+^ to NADH (Figure5a). The absorbance or fluorescence of NADH can then be used to follow the reaction. We find that fluorescence, as used by Dunn *et al.* (2013), to be the most convenient signal because it is straightforward to scale the assay format to smaller cuvette volumes in order to conserve enzyme.

Figure5b shows an example of raw assay data from an experiment carried out at 10 μm octanoyl-CoA, 0.125% (21.4 mm) ethanol and 0.14 μm Eht1. Initial rates (*v_0_*) could be determined from these enzyme progress curves by linear curve fitting. Unexpectedly, the reaction was not sensitive to ethanol concentration, as would be expected of one of the substrates in a bisubstrate transferase reaction. Increasing ethanol concentrations actually poisoned the reaction (not shown), presumably by destabilizing Eht1 or another assay component. The assay buffer contained 7% glycerol from the *α*-ketoglutarate dehydrogenase storage buffer but gel-purifying the dehydrogenase had no effect on activity (not shown), suggesting that this glycerol does not play a role in the reaction.

It thus appears that, as well as an alcohol acyltransferase, Eht1 can also act as a thioesterase, generating medium-chain fatty acids from acyl-CoAs even at high ethanol concentrations. Comparative sequence analysis suggests that the active site of Eht1 features a Ser^247^–Asp^395^–His^423^ catalytic triad (Saerens *et al.*, 2006). This was confirmed experimentally by site-directed mutagenesis, with Eht1 activity in the coupled assay being abolished by the active site mutations S247A and D395N (Figure5b).

**Figure 6:**
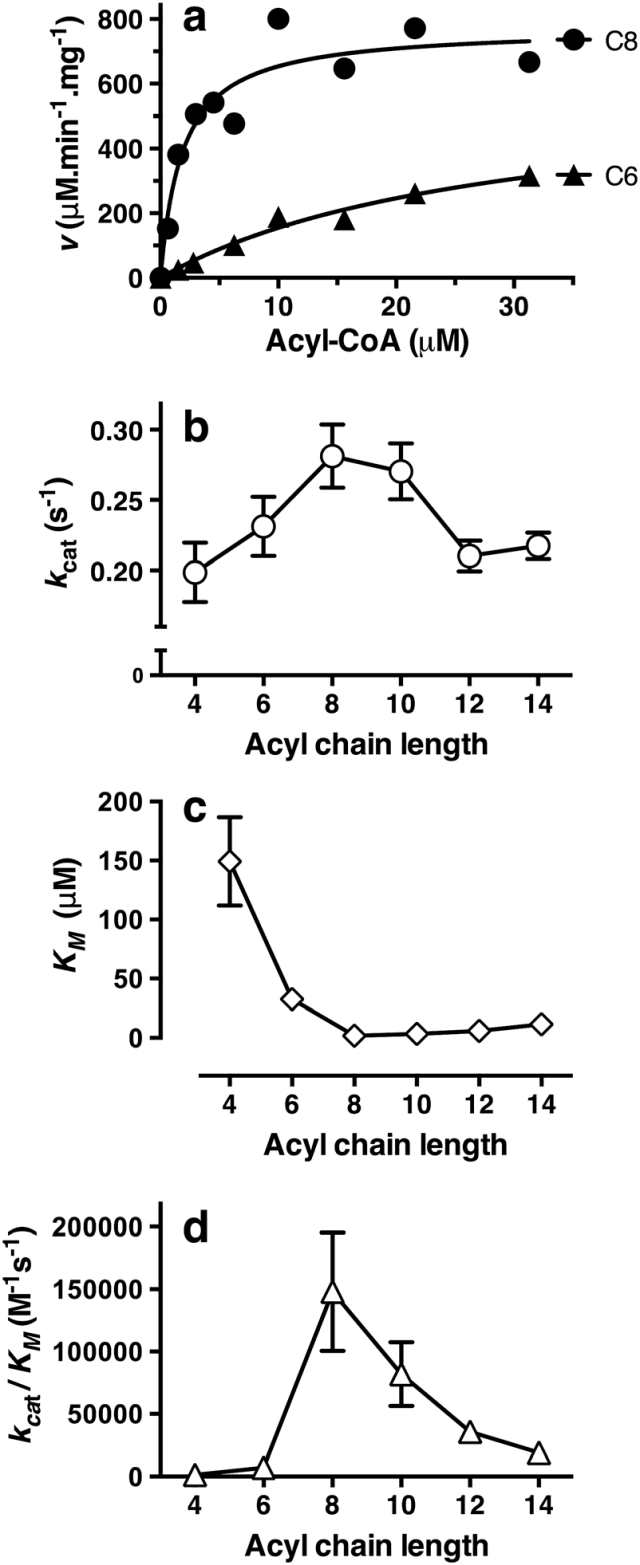
Acyltransferase activity of recombinant Eht1. (a) Coupled enzyme assays displayed hyperbolic Michaelis–Menten kinetics. C6, hexanoyl-CoA; C8, octanoyl-CoA. (b) turnover number, (c) substrate binding affinity and (d) catalytic efficiency were optimal for octanoyl-CoA. Data are ± SE from non-linear regression

The activity of Eht1 toward acyl-CoA substrates of chain length C4–C14 followed classical Michaelis–Menten kinetics (Figure6) with rates two orders of magnitude greater than previously seen for recombinant Eht1 isolated from *E. coli* (Saerens *et al.*, 2006). We do not know the oligomeric state of Eht1 in these reactions, but assume that the monomer is the active unit. Unexpectedly, the preferred substrate for Eht1 was octanoyl-CoA (largest *k_cat_*/*K_M_*) with *k_cat_* = 0.28 ± 0.02/s and *K_M_* = 1.9 ± 0.6 μm. The enzyme reaction rate was systematically diminished at shorter or longer chain lengths and the same trend in reaction rate was also observed in recombinant membrane extracts prior to purification (not shown). Substrate affinity (lowest *K_M_*) increased sharply up to C8 and then decreased marginally at longer chain lengths.

### Esterase activity of Eht1

**Figure 7:**
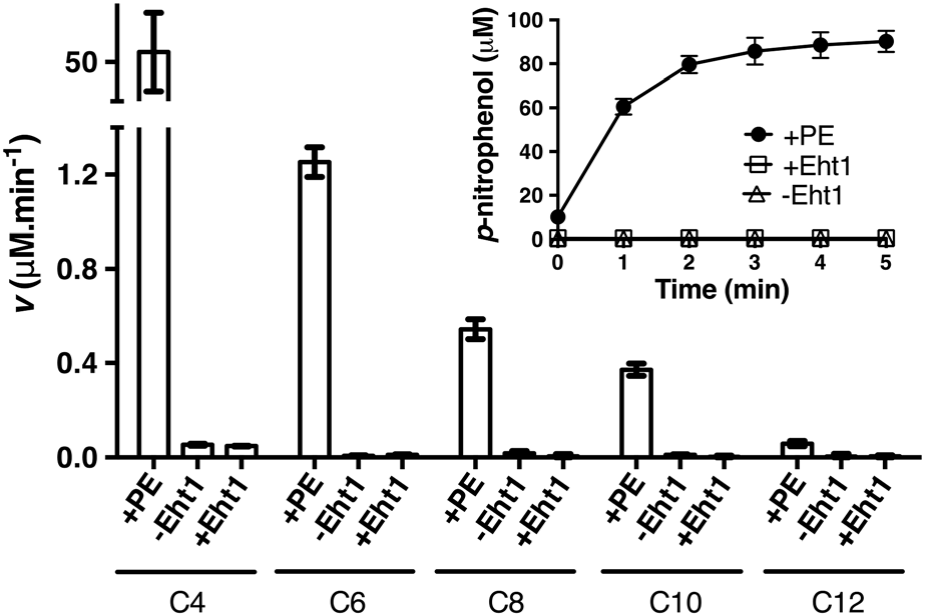
Esterase activity of recombinant EHT1. No esterase activity was observed relative to a positive control (porcine esterase, PE) or negative control (–Eht1) at any of the acyl chain lengths tested. Inset graph shows raw data for *p*-nitrophenyl butyrate. Data are mean ± SD from three replicates

The proposed esterase activity of Eht1 (Saerens *et al.*, 2006) was assessed using *p*-nitrophenol esters with acyl chain lengths C4–C12 (Figure7). No activity was observed relative to a positive control with a porcine liver esterase, or to a negative control without enzyme. These data contradict previous findings (Saerens *et al.*, 2006) that Eht1 can act as a homeostatic esterase.

## Discussion

FAEEs are produced by higher organisms in response to ethanol consumption (Zelner *et al.*, 2013), as secondary metabolites in yeast and fungi during fermentation (Mason and Dufour, 2000; Saerens *et al.*, 2010) and in plants during fruit ripening (El Hadi *et al.*, 2013). Volatile short- and medium-chain length (C2–C12) FAEEs make a significant contribution to the flavour profile of fruits and of industrial fermented beverages such as beer and wine. Medium-chain FAEEs are synthesized enzymatically by specific acyltransferases, but the details of this process are not well appreciated. Understanding the biochemistry of yeast and plant alcohol acyltransferases may lead to designer yeast strains with tailored fermentation products (Pretorius *et al.*, 2003; Lilly *et al.*, 2006; Chen *et al.*, 2014), enable improvements to the flavour characteristics of certain fruit by selective breeding or genetic modification (El Hadi *et al.*, 2013) and allow for the development of engineered yeast and bacteria that produce renewable biofuels and fine chemicals (Shi *et al.*, 2012; de Jong *et al.*, 2014; Rodriguez *et al.*, 2014; Runguphan and Keasling, 2014).

Saerens *et al.* (2006) provided the first evidence that studying recombinant AEATases *in vitro* could offer useful insights into the biochemistry of these proteins. Here, we build upon that work by recombinantly expressing his-tagged yeast AEATases within a yeast host. Appropriate localization and post-translational processing remain significant obstacles to expressing eukaryotic membrane proteins in prokaryotic systems (Freigassner *et al.*, 2009; Schlegel *et al.*, 2010) and the major advantage of our approach is that the expressed protein can be targeted to, and purified directly from, the sedimenting lipid fraction within which it natively resides. Using this approach, we purified Eht1 to homogeneity at the milligram scale in a single affinity chromatography step. Eeb1 and Ymr210w cannot be expressed using this system, suggesting that the cellular abundance of these proteins is tightly controlled. It may be possible in future to avoid transcriptional regulation by expressing a synthetic *Eeb1* gene with a novel DNA sequence that codes for the same protein sequence. Ymr210w also appears to be regulated post-translationally (Figure1) and so is likely to prove generally recalcitrant to yeast overexpression. As an alternative approach, cell-free expression systems should be considered as a means of producing Eeb1 and Ymr210w.

A continuous coupled enzyme assay was introduced to characterize the substrate preference and enzyme kinetics of recombinant Eht1 (Figures5, 6). The preferred substrate for Eht1 is octanoyl-CoA (highest *k_cat_*/*K_M_*). The data from these experiments are consistent with a model in which substrate binding is dictated by the length of the substrate acyl chain. Eht1 showed the highest affinity (lowest *K_M_*) toward octanoyl-CoA and substrate affinity was only slightly reduced at increasing acyl chain lengths up to C14. However, the enzyme activity was diminished at acyl chain lengths > C8, presumably either because of slower product off-rates or because of suboptimal disposition of the substrate within the active site. The preference towards C8 acyl chains determined by this assay, which measures the generation of free CoA, are in agreement with data from qualitative GC–MS that characterizes volatile products (Figure4). Our data also provide strong experimental support for the presence of the proposed *α*/*β*-hydrolase functional domain with a Ser–Asp–His catalytic triad.

The enzyme kinetics of yeast and fruit alcohol acyltransferases have, to date, generally been studied by single time-point enzyme assays. These assays characterize the reaction products with quantitative GC–MS, determine free CoA with 5,5′-dithiobis-(2-nitrobenzoic acid) (DTNB) or use radiolabelled substrates (Pérez *et al.*, 1996; Aharoni *et al.*, 2000; Lilly *et al.*, 2000; Olías *et al.*, 2002; Verstrepen *et al.*, 2003; Beekwilder *et al.*, 2004; El-Sharkawy *et al.*, 2005; Saerens *et al.*, 2006; Luchetta *et al.*, 2007; Balbontín *et al.*, 2010; Gunther *et al.*, 2011; Chen *et al.*, 2014). The coupled assay introduced here is advantageous because it allows the measurement of the initial (linear) enzyme rate at each substrate concentration and avoids some of the drawbacks of single time-point assays (assumptions of linear reaction rate and substrate excess for all substrate concentrations at that specified time point). We anticipate that this assay could be readily applied to study the functions of other alcohol acyltransferases.

Unexpectedly, we found that Eht1 was also active as a thioesterase and could hydrolyse medium-chain acyl-CoAs to generate free fatty acids (Figure5). However, Eht1 does not appear to act as a generic hydrolase, since it was unable to hydrolyse *p*-nitrophenyl acyl esters. These results support previous suggestions (Bardi *et al.*, 1998; Saerens *et al.*, 2010) that the primary metabolic role of the AEATases is to recover free CoA from medium-chain acyl-CoAs that accumulate in the yeast cell under anaerobic conditions. Both free fatty acids and ethanol can inhibit fermentation and cell growth and Eht1 may be sequestered within the lipid particle/mitochondria in order to promote protein interactions with acyl-CoAs and ethanol. This could favour the synthesis of FAEEs to detoxify fatty acids and ethanol.

We thus present here the characterization of Eht1, a yeast AEATase with an important role in industrial fermentation. This study reveals several unexpected properties of Eht1, particularly that this enzyme is specific for octanoyl-CoA and can act as a thioesterase as well as an alcohol acyltransferase. Further work will be needed to explore the structural basis for substrate selectivity and determine whether the methods described here can be applied to other acyltransferases from yeast and plants.
